# Effect of Various Mulberry Leaf Powders on the Quality of Artificial Diet for Domestic Silkworm, Bombyx mori

**DOI:** 10.3390/insects17060538

**Published:** 2026-05-22

**Authors:** Ke Xu, Yang Gui, Xinxin Zuo, Han Chen, Zhiqing Li, Ping Lin, Guanwang Shen

**Affiliations:** Integrative Science Center of Germplasm Creation in Western China (Chongqing) Science City, Biological Science Research Center, Southwest University, Chongqing 400715, Chinalizhiqing@swu.edu.cn (Z.L.); linpingswu@swu.edu.cn (P.L.)

**Keywords:** mulberry variety, mulberry leaf powder, artificial diet quality, silkworm rearing, metabolomics

## Abstract

This study aimed to investigate how mulberry leaf powders from different mulberry varieties influenced the quality of artificial diet for domestic silkworms. Using metabolomics, we examined the possible reasons why silkworm rearing performance varied, and provided new research ideas for improving diet quality. Forage mulberry leaves differed from grafted mulberry leaves in metabolic pathways related to fatty acids, amino acids, and vitamin C. Forage mulberry leaf powder showed considerably decreased amounts of vitamin C, serine, linoleic acid, and linolenic acid, all of which are required for the production of silk proteins and their intermediates. A considerable increase in 1-deoxynojirimycin (DNJ) content was observed. The feeding characteristics of silkworms led to decreased DNJ tolerance and lower quality of silkworm diet made from forage mulberry leaf powder compared to that made from grafted mulberry leaf powder. Furthermore, the body weight during the silk-spinning stage, cocoon weight, and Cocoon shell ratio of the reared silkworms were all markedly reduced. Using mulberry leaf powder would require addressing the problem of reduced DNJ tolerance in silkworms owing to the changes in their diet, in addition to improving the composition of the silkworm diet based on the characteristics of the mulberry leaf powder.

## Simple Summary

Silkworms are economically crucial insect species. The use of artificial silkworm diet is critical for the expansion and automation of the sericulture sector. This study used metabolomics to investigate the metabolite differences between two types of mulberry leaves to evaluate the effects of different mulberry leaf powder sources on the quality of the artificial silkworm diet. Furthermore, the performance of silkworms fed an artificial diet composed of these mulberry leaf powder was assessed. The forage mulberry leaves contained considerably lower quantities of serine, linoleic acid, linolenic acid, its intermediates, and vitamin C than the grafted mulberry leaves. Contrastingly, the forage mulberry leaves had a much greater 1-deoxynojirimycin concentration. Silkworms fed artificial diet derived from these leaves exhibited reduced weight, as well as decreased cocoon weight and Cocoon shell ratio. Thus, the different metabolite contents of the two mulberry varieties may have led to various silkworm-rearing outcomes. This provides guidelines and scientific evidence for choosing appropriate mulberry types and adjusting the composition of artificial silkworm diet.

## 1. Introduction

Mulberry trees (*Morus alba* L.) are the foundation of the sericulture industry. In addition to its medicinal properties, its leaves are mostly utilized to feed domestic silkworms (*Bombyx mori*) [1]. However, the conventional method of raising silkworms is highly dependent on fresh mulberry leaves, which leads to severe seasonality, high labor costs, and small production scales. Although artificial diet for all stages of the silkworm life was successfully developed in 1960 [2], this technology has not yet been widely used. In recent years, the benefits of artificial diet rearing have become more obvious as labor and land resources have become more limited. It frees silkworm farming from relying on fresh leaves, avoids competition with agriculture for time and land, and establishes conditions for large-scale intelligent farming.

The composition [3] and processing technology [4] of artificial silkworm diet have considerably advanced in recent years; however, the high cost of the diet continues to be a major barrier to the industry’s growth. The price of mulberry leaf powder markedly influences diet production costs because it is the primary raw material with the highest unit cost in artificial diet [4]. Consequently, lowering the price of mulberry leaf powder has emerged as a key development for advancing artificial diet industrialization for silkworm rearing.

Grafted mulberry varieties currently grown in China, such as Qiang Sang No. 1, have thick trunks and a high degree of lignification [5], making mechanical harvesting difficult and maintaining high labor costs, even though they produce high-quality leaves. Contrastingly, new hybrid forage mulberry cultivars, including Yue Sang No. 11, are bushy shrubs with minimal lignification that work well with the forage harvesting equipment currently in use. They can be harvested three to six times a year [6,7], which substantially lowers the harvesting cost. Diet prices would probably markedly drop if forage mulberry leaf powder replaced grafted mulberry leaf powder as a raw material for artificial diet.

Nevertheless, mulberry leaves have been used as a food source for domestic silkworms for thousands of years. The mulberry leaf composition and nutritional requirements of the silkworm for growth and development have co-evolved to almost perfect agreement. Mulberry leaves are the primary ingredient in almost all diets utilized to raise silkworms; other ingredients are only used to compensate for nutrients that are missing due to reduced mulberry leaf content [8]. Notable variations exist in the nutritional components of various mulberry types, including moisture, crude protein, amino acids, crude fat, and soluble sugars [9], as well as in the efficacy of silkworm rearing when using fresh leaves [10]. Thus, determining if mulberry leaf powder from forage mulberry is appropriate for artificial silkworm diet and compensating for its nutritional deficits are urgently required. This study aimed to ascertain whether the quality of artificial diet for domestic silkworms was affected by mulberry leaf powder obtained from two varieties, Yue Sang No. 11 (forage mulberry) and Qiang Sang No. 1 (grafted mulberry). Based on this, we used metabolomics to investigate the differences in leaf metabolites between the two varieties and assessed the viability of using forage mulberry leaves as a replacement for grafted mulberry leaves in artificial diet for domestic silkworms. Additionally, we provide scientific evidence and recommendations for selecting mulberry varieties for silkworm diet and optimizing silkworm diet formulations.

## 2. Materials and Methods

### 2.1. Materials

The Chongqing Academy of Sericulture supplied the four-way hybrid silkworm strain Liangguang No. 2. In April 2024, leaves of forage mulberries (Yue Sang No. 11, code S7) and grafted mulberries (Qiang Sang No. 1, code S3) were collected from the Yongchuan District of Chongqing. Leaves from trees free of pests and diseases and with steady growth vigor were chosen, and leaves of the same variety were evenly combined. A portion of the mulberry leaves was immediately flash-frozen with liquid nitrogen and kept at −80 °C for further metabolomics research, whereas another fraction was dried and pulverized to create artificial silkworm diet. The Biology Research Center of Southwest University supplied the artificial silkworm meal. Table 1 lists the main components.

### 2.2. Artificial Diet Processing and Silkworm Rearing Methods

To 100 g of the prepared artificial silkworm diet, 200 mL of distilled water was added and stirred until they combine well. The mixture was subsequently steamed in water. Furthermore, the mixture was cooled to room temperature after steaming and stored at 4 °C. The temperature of the mixture was then brought back to room temperature before using. Once the silkworms hatched, they were gathered, placed on the diet, and raised in an incubator. The temperatures were as follows: First instar: 29 ± 1 °C and 80 ± 5% humidity; Stages 2–3: 27 °C and 75 ± 5% humidity; Stage 4: 25 °C and 70 ± 5% humidity; Stage 5: 24 °C and 70 ± 5% humidity; and light: dark cycle as 12L:12D. After each molt, any remaining diet and silage were removed from instars 1 to 3 and were replaced with an equivalent quantity of fresh diet. For instars 4 to 5, the leftover diet and silage were removed and replaced with an equivalent amount daily. The body weight, total cocoon weight, cocoon shell weight, and Cocoon shell ratio (calculated as: (cocoon shell weight/total cocoon weight) × 100%) of silkworms fed the various diets (S3 and S7) were measured during the same developmental phase. Three parallel experiments were conducted with *n* = 30.

### 2.3. Ultra-High Performance Liquid Chromatography-Tandem Mass Spectrometry (UPLC-MS/MS)

Metabolite data were collected using an ultra-high-performance liquid chromatography system (Nexera X2; Shimadzu Corporation, Kyoto, Japan) and a tandem mass spectrometry system (4500 QTRAP; Applied Biosystems, Waltham, MA, USA). An Agilent column (SB-C18, 1.8 µm, 2.1 mm × 100 mm) was used. The mobile phases were ultrapure water (containing 0.1% formic acid) and acetonitrile (containing 0.1% formic acid) at a 0.35 mL/min flow rate. The column temperature was 40 °C, and 4 μL of the sample was injected for a single analysis. The eluate was alternately connected to an electrospray ionization (ESI)-triple quadrupole linear ion trap AB4500 platform (QTRAP UPLC/MS/MS, SCIEX, Darmstadt, Germany) for analysis. The ion source (source temperature 550 °C) was set to turbo spray, with the ion spray voltage set to 5500 V (positive ion mode), and −4500 V (negative ion mode). The nebulizer gas (GSI), auxiliary gas (GSII), and curtain gas (CUR) were set to 50, 60, and 25 psi, respectively, and the system was operated under high-energy collision-induced ionization.

### 2.4. Data Processing

#### 2.4.1. Metabolomics Data Processing and Statistical Analysis

Analyst 1.6.3 was utilized to process the spectral data, and secondary spectral information from the Metware Database was used to qualitatively characterize the materials (MWDB, Metware Corporation, Wuhan, China). During the study, the fragmentation signals from other macromolecules and isotope signals from K^+^, Na^+^, and NH_4_^+^ were eliminated. Multiple reaction monitoring (MRM) was employed to quantify the metabolites. The relative abundance of each metabolite was ascertained by calculating the peak areas after the chromatographic peaks were adjusted using MultiaQuant 3.0.

#### 2.4.2. Principal Component Analysis (PCA) and Partial Least Squares Discriminant Analysis

The statistical function prcomp in R (version 3.5.1) was used to perform unit-variance scaling and unsupervised PCA of the data. The variance of each principal component was calculated to evaluate its contribution to explaining the model’s structure. The PCA model was validated using Partial Least Squares Discriminant Analysis (PLS-DA), and Variable Importance Projection (VIP) values for each metabolite were determined.

#### 2.4.3. Systematic Clustering and Pearson Correlation Coefficients

The R package ComplexHeatmap 2.8.0 was utilized for clustering analysis and heatmap visualization of the samples and metabolites. Hmisc version 4.4.0 was used to calculate the Pearson’s correlation coefficients between the samples.

#### 2.4.4. Differential Metabolite Identification

Based on the criteria where VIP ≥ 1 and the absolute logarithm of Fc (multiplicative change) ≥ 1, metabolites showing considerable differences across groups were identified. VIP values were extracted from the OPLS-DA data using the R package MetabAnalystR 1.0.1 in univariate analysis. Ranking charts and scoring plots were created to identify differences in the metabolites between groups. Log2FC values were used to further identify metabolites. The metabolites were divided into three groups according to their tandem mass spectrometry and reference database-matching scores. The most promising differentially expressed metabolites were identified using this method.

#### 2.4.5. Kyoto Encyclopedia of Genes and Genomes (KEGG) Annotation and Enrichment Analysis

Identified metabolites were annotated using the KEGG compound database. Annotated metabolites were subsequently mapped to the KEGG Pathway database. Next, the pathways containing metabolites with substantial regulatory effects were selected for Metabolite Set Enrichment Analysis (MSEA). Their significance was determined based on the *p*-values obtained from hypergeometric tests. Differential abundance (DA) scores were calculated to capture the overall changes in all differential metabolites (DMs) within each pathway.

### 2.5. Detection of the DNJ Content

#### 2.5.1. Materials and Solvents

A cold, dry location was utilized to maintain the crushed and dried mulberry leaves. The Biology Research Center at Southwest University supplied an artificial meal for the silkworms. Shanghai Yuanye Biotechnology Co., Ltd. (Shanghai, China) supplied the reference substance of 1-deoxynojirimycin (DNJ) (CAS number: 19130-96-2, purity ≥ 98%). Thermo Fisher Scientific Co., Ltd. (China), (Shanghai, China) supplied acetonitrile (chromatographic grade), and Shanghai Aladdin Biochemical Technology Co., Ltd. (Shanghai, China) supplied acetic acid (chromatographic grade) and ammonium acetate (chromatographic grade).

#### 2.5.2. Equipment and Methods

A Vanquish Flex ultra-high-performance liquid chromatography (UHPLC) system (Thermo Scientific Vanquish Flex UHPLC, Thermo Fisher Scientific (China) Co., Ltd., China) with an Alltech 3300 evaporative light-scattering detector (ELSD, Alltech, Inc., Deerfield, IL, USA) was used.

#### 2.5.3. Preparing the Sample

A 7-fold volume of 80% ethanol-water solution was added to every gram of mulberry leaf powder or diet powder. Using ultrasonic-assisted extraction, DNJ was extracted from the mulberry leaves. The ultrasonic treatment lasted 260 s, the water bath temperature was kept at 40 °C, and the ultrasonic power was set at 180 W. Subsequently, the mixture was centrifuged for 10 min at 11,500× *g* and 4 °C. The supernatant was collected, vacuum evaporated, and dissolved in ultrapure water.

#### 2.5.4. Conditions for High-Performance Liquid Chromatography (HPLC)

A Yuexu Ultimate HILIC-Amide column (4.6 × 250 mm, 5 μm; Yuexu Technology (Shanghai) Co., Ltd., Shanghai, China) was used and 40 °C was used as the column temperature. Mobile phase B included acetonitrile and mobile phase A included 10 mM aqueous ammonium acetate solution (pH 5.5 adjusted with acetic acid). Program for gradient elution included the following: 0–15 min: 85% B; 15–25 min: 70% B; 25–26 min: 65% B; and 26–36 min: 85% B. The ELSD drift tube temperature was 55 °C, the flow rate was 1.0 mL/min, the injection volume was 10 μL, and the carrier gas flow rate was 1.6 SLM.

### 2.6. Statistical Analysis

Microsoft Excel was used to organize the raw experimental data and compute variance and mean values. Differences between the two groups were evaluated using the independent samples *t*-test. Data are presented as the mean ± standard error of the mean (SEM). A *p*-value of less than 0.05 was considered statistically significant.

## 3. Results

### 3.1. Evaluation of the Silkworm Rearing Efficiency Using Two Kinds of Mulberry Leaf Powder as Raw Materials

**Figure 1:**
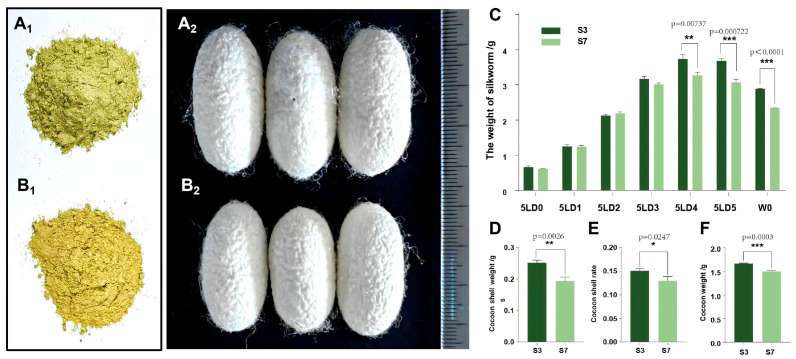
Growth performance and cocoon traits of silkworms fed artificial diets containing mulberry leaf powder from two different varieties. (**A_1_**,**A_2_**): Diet prepared from mulberry leaf powder from grafted mulberry trees, and the cocoons obtained from rearing silkworms using this diet; (**B_1_**,**B_2_**): Diet prepared from mulberry leaf powder of the forage mulberry, and the cocoons obtained from rearing silkworms on this diet; (**C**–**F**): Weight/Cocoon shell weight/Cocoon shell ratio (Cocoon shell weight/Cocoon weight)/Cocoon weight (Cocoon shell weight + Pupa) of the silkworms reared on diet prepared from mulberry leaf powder from different mulberry trees; 5LD0/1/2/3/4/5: Silkworms at 0/1/2/3/4/5 days of their fifth instar, W0: Mature silkworms (have stopped feeding and are preparing to spin cocoons), S3: Diet made from mulberry leaf powder from grafted mulberry trees, S7: Diet made from mulberry leaf powder of the forage mulberry. Data were expressed as mean ± SEM. Differences in data were assessed by Student’s *t*-test, * *p* < 0.05; ** *p* < 0.01; *** *p* < 0.001.

The color of the mulberry leaf powder-based S3 (Qiang Sang No. 1) diet (Figure 1A_1_) was darker than that of the S7 (Yue Sang No. 11) diet (Figure 1B_1_). Compared with silkworms fed S7 (Figure 1B_2_), those fed S3 had larger cocoons (Figure 1A_2_). The body weight of silkworms fed S3 was considerably higher than that of silkworms fed S7, according to an analysis of body weight changes and cocoon quality attributes, after reaching the peak feeding phase (5LD4) (Figure 1C). Furthermore, silkworms fed the S3 diet had substantially higher cocoon shell weight (Figure 1D), Cocoon shell ratio (Figure 1E), and total cocoon weight (Figure 1F) than those fed the S7 diet. In conclusion, the artificial diet made with mulberry leaf powder from grafted mulberry trees (Qiang Sang No. 1, S3) performed better than the artificial diet made with mulberry leaf powder from forage mulberry trees (Yue Sang No. 11, S7). This suggests that the source of mulberry leaf powder affects the capacity of the silkworm to adapt to the meal.

### 3.2. Comparison of the Metabolite Differences of the Two Mulberry Species’ Leaf Powder

**Figure 2:**
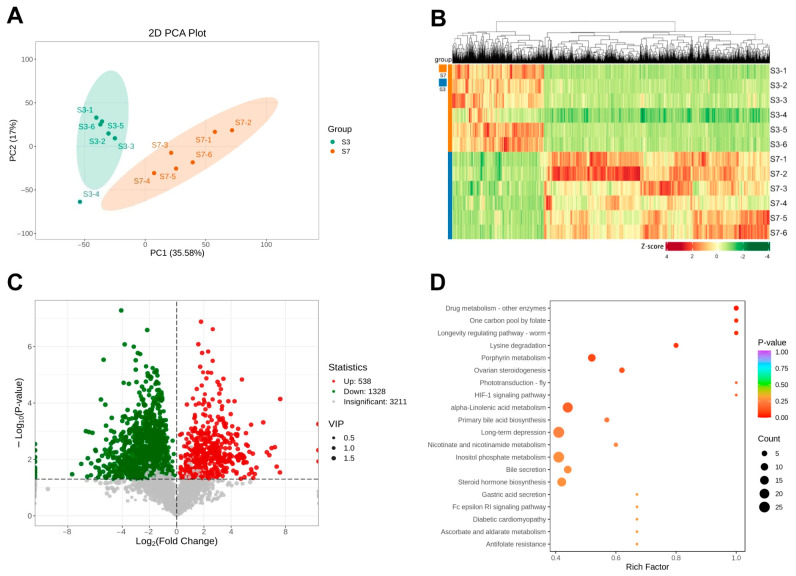
Analysis of the differential metabolites: (**A**): Principal component analysis of mulberry leaf samples from the two varieties. (**B**): Heat map of the differential metabolites in the mulberry leaves from the two varieties. (**C**): Volcano plot of the differential metabolites in the mulberry leaves from the two varieties. (**D**): Kyoto Encyclopedia of Genes and Genomes enrichment analysis of the differential metabolites in the mulberry leaves from the two varieties.

We utilized metabolomics to assess the metabolic differences between the two mulberry leaf types to determine the causes of substantial differences in the silkworm rearing performance between diets generated from various mulberry leaf types. The S3 and S7 groups were completely separated according to PCA (Figure 2A), suggesting that their metabolic profiles significantly differed. A total of 1866 differentially expressed metabolites, comprising 538 upregulated and 1328 downregulated metabolites, were found by combining univariate *p*-values and fold change (FC) values under the criteria of VIP > 1.0, and *p*-values < 0.05 (Figure 2B,C). The differentially expressed metabolites were considerably enriched in the pathways linked to vitamin, sterol, and glycolipid metabolism, according to KEGG analysis (Figure 2D).

### 3.3. Differential Metabolite Functional Analysis

To assess the potential reasons for the influence of various varieties of mulberry leaf powder on silkworm rearing performance, we concentrated on the changes in the levels of pertinent compounds within several metabolic pathways that are closely associated with domestic silkworm growth and development (Table 2).

**Figure 3:**
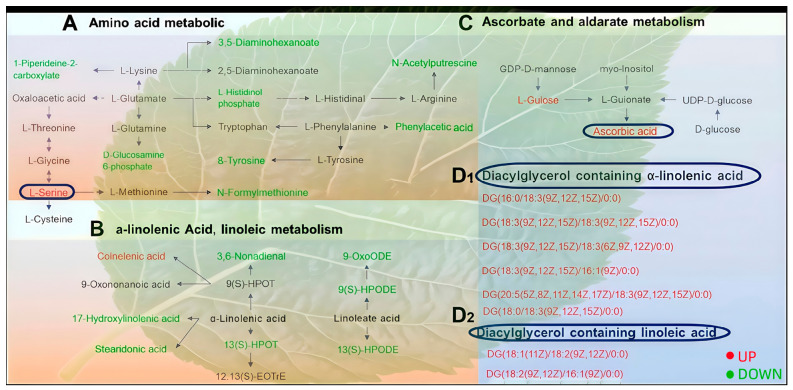
Synthesis pathways of the key differential metabolites: (**A**): Amino acid metabolic pathway. (**B**): Metabolism of α-Linolenic acid and linoleic acid. (**C**): Vitamin C metabolism; (**D_1_**): Diacylglycerol containing α-Linolenic acid; (**D_2_**): Diacylglycerol containing linoleic acid; Red indicates up-regulated metabolites; green indicates down-regulated metabolites (relative to the S7 diet group).

First, grafted mulberry leaves had a higher (1.53-fold) serine content than the forage mulberry leaves, whereas other amino acids were not directly detected. Forage mulberry leaves generally exhibited higher levels of their downstream products (Figure 3A). Second, linoleic acid and α-linolenic acid are mostly found in mulberry leaves as glycerides, and silkworms are unable to produce these compounds on their own. Metabolites associated with the metabolic pathways of α-linolenic acid and linoleic acid were generally increased in the leaves of forage mulberry plants (Figure 3B). Furthermore, the content of diglycerides composed of α-linolenic acid (Figure 3D_1_) and linoleic acid (Figure 3D_2_) was substantially lower in the forage mulberry leaves compared to the grafted mulberry leaves. Thus, the actual available amounts of α-linolenic and linoleic acids in the leaves of forage mulberry plants may be low.

Additionally, the content of α-linolenic acid-containing diglycerides in the grafted mulberry leaf powder was generally higher than that in the forage mulberry leaf powder. Furthermore, the differential metabolite content in the α-linolenic acid degradation pathway was markedly lower in the grafted mulberry leaf powder than in the forage mulberry leaf powder. The two mulberry leaf types demonstrated considerable differences in the levels of vitamins and certain fatty acids that are necessary for silkworm growth and development, but cannot be synthesized by the silkworms themselves. In particular, the vitamin C content of the forage mulberry was significantly lower than that of the grafted mulberry (Figure 3C).

**Figure 4:**
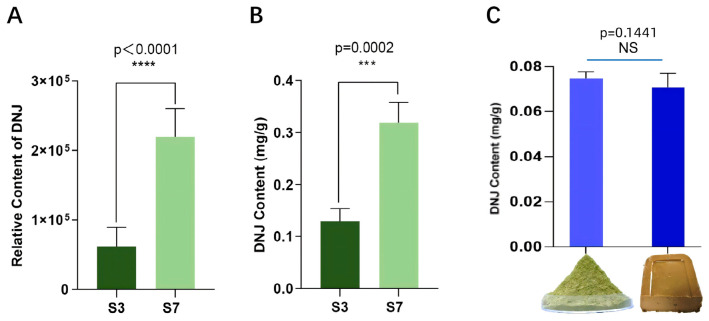
Detection of the 1-deoxynojirimycin (DNJ) content in mulberry leaves and diet. (**A**): The relative content of DNJ in mulberry leaf powders from the two varieties; (**B**): The content of DNJ in the mulberry leaf powders from the two varieties determined using high-performance liquid chromatography; (**C**): The DNJ content in the diet before and after maturation. Data were expressed as mean ± SEM. Differences in data were assessed by Student’s *t*-test; *** *p* < 0.001; **** *p* < 0.0001; NS *p* > 0.05.

According to metabolomic research, forage mulberry (S7) had a much greater relative concentration of DNJ than grafted mulberry (S3) (Figure 4A). We speculate that DNJ may be one of the primary causes of the lower silkworm-rearing performance of diet made with forage mulberry leaf powder because it has a substantial inhibitory influence on insect growth and development. To confirm this hypothesis, we used HPLC to quantitatively analyze the DNJ content in the two mulberry leaf powders. In line with the metabolomic results, the DNJ concentration in the forage mulberry leaf powder was much higher than that in the grafted mulberry leaf powder (Figure 4B). Considering that the artificial diet underwent a conditioning process during processing, which may have affected the DNJ content, we further tested the DNJ content in the artificial diet before and after conditioning. No significant difference was noted in the DNJ content before and after conditioning (Figure 4C). This indicated that DNJ was stable during diet processing and that its content was mainly determined by the raw material itself, rather than by the processing method.

In conclusion, the concentrations of metabolites, including vitamins, essential fatty acids, and amino acids, substantially varied among the mulberry leaf varieties. When domestic silkworms are fed artificial diets made with matching mulberry leaf powder as a raw material, these variations may have an overall effect on their growth, development, and the silk protein synthesis.

## 4. Discussion

Metabolomics is a biological tool for evaluating metabolic responses to environmental alterations, bridging the genotype–phenotype gap present in transcriptomics and proteomics [11]. It is widely used to study the accumulation of naturally active metabolites in various tissues of mulberry trees, the molecular basis of mulberry tree adaptation to abiotic stress, the development of naturally active metabolites [12], and the response of domestic silkworms to artificial diets [13]. Based on the finding that significant differences exist in the performance of silkworm rearing between artificial diets prepared from grafted mulberry leaves and those prepared from forage mulberry leaf powder, this study employed metabolomics to compare the metabolic differences between the two mulberry leaf varieties. This study aimed to identify substances in mulberry leaves that may influence the quality of artificial silkworm diet and explore potential improvement methods.

In addition to being parts of proteins, amino acids also function as signaling molecules. They regulate intercellular communication, protein phosphorylation, gene expression, and food intake [14]. Previous studies have shown that amino acids such as tyrosine [15] and valine [16] have major effects on sericin protein synthesis. We discovered that even though grafted mulberry leaves demonstrated a far lower relative concentration of these amino acid derivatives than forage mulberry leaves, the diet made with the grafted mulberry leaf powder performed better in growing silkworms than forage mulberry. This could be explained by an imbalance in the amino acid ratio in the meals made from the two varieties of mulberry leaf powder. Unbalanced amino acid ratios in artificial silkworm diets diminishes DNA replication, lower sericin-related gene expression, and cause mild autophagy in the posterior silk glands, which decreases the weight and proportion of the cocoon shell [17].

Linoleic and alpha-linolenic acids are essential dietary fatty acids for domestic silkworms. According to previous research, polyunsaturated fatty acids (like linoleic acid and alpha-linolenic acid) can activate the hypothalamic fatty acid-sensing mechanism of mandarin fish, control the gene expression of neuropeptides associated with appetite, and ultimately affect feeding behavior [18]. Moreover the addition of 4% alpha-linolenic acid to the diet promotes the growth of *Apis mellifara meda* [19]. Consequently, the relative lack of these two essential fatty acids in mulberry diet may affect the physiological state and feeding motivation of silkworms, leading to reduced rearing performance. Thus, the increased α-linolenic acid concentration of diet prepared from grafted mulberry leaf powder may be associated with the enhanced silkworm rearing performance reported.

Vitamin C, as an important intracellular water-soluble antioxidant, is essential for immunological control and oxidative stress resistance [20]. A sufficient amount of vitamin C stimulates silk protein synthesis in silkworms. Contrastingly, a vitamin C deficit can cause stunted growth and even mortality, according to previous research [21,22]. Consequently, the low vitamin C concentration in forage mulberry could be a dietary constraint affecting how well silkworms are raised.

DNJ has hypoglycemic, antiviral, and anticancer properties and is a distinctive component of the polyhydroxy alkaloids found in mulberry leaves. It is mostly extracted from the branches, leaves, and roots [23]. A recent study identified that *Pseudomonas fulva* in the silkworm gut can efficiently degrade DNJ, a mulberry defense toxin, highlighting its role as a key symbiotic bacterium for host tolerance [24]. However, 16S rRNA sequencing data from Dong et al. revealed significant alterations in the gut microbiota of silkworms reared on an artificial diet, showing that the abundance of the genus *Pseudomonas* was substantially reduced compared to that in the mulberry leaf-fed group [25]. This diet-driven dysbiosis may impairs the colonization of DNJ-degrading *Pseudomonas* strains (e.g., *P. fulva*), thereby compromising the host’s ability to detoxify DNJ. Excess DNJ from mulberry leaves in the diet substantially inhibits silkworm growth and development [26]. The DNJ content of Yue Sang No. 11 was substantially higher than that of Qiang Sang No. 1, which may be another crucial reason for its poor silkworm-rearing performance.

## 5. Conclusions

Thus, compared to the grafted mulberry, differences in the metabolic pathways of amino acids, fatty acids, and vitamin C in forage mulberry led to decreased levels of serine, linoleic acid, and linolenic acid, as well as their intermediates and vitamin C, in the corresponding mulberry leaf powder. These are necessary for the synthesis of silk protein; however, the DNJ content increased. Combined with the decreased tolerance of silkworms to DNJ owing to the characteristics of artificial silkworm diet, this leads to artificial silkworm diet made from forage mulberry leaf powder having a lower quality than that made from grafted mulberry leaf powder. Thus, making targeted enhancements to the silkworm diet composition based on the forage mulberry leaf powder’s characteristics and addressing the issue of reduced DNJ tolerance in silkworms caused by alterations in their gut microbiota resulting from the diet will be necessary if mulberry leaf powder from forage mulberry is used as a raw material in the future.

## Figures and Tables

**Table 1:** Composition of the artificial diet.

Constituent	Content/%
Soybean meal	40
Corn flour	10
Vitamin and Mineral Premix	3
Binder and Other Additives	17
Mulberry leaf powder	30
Total	100

**Table 2:** Key differential metabolites.

Metabolic Pathway	Index	Compounds	Class II	*p*-Value	Fold_ Change	Type
Amino acid metabolic	MW0140737	1-piperidine-2-carboxylate	Organic acid and Its derivatives	0.0016	0.16	down
	MW0109669	L-Serine	Amino acids	0.0222	1.53	up
	MW0114223	D-glucosamine 6-phosphate	Organic acid and Its derivatives	0.0024	0.13	down
	MW0103913	(3S,5S)-3,5-Diaminohexanoate	Esters	0.0117	0.08	down
	MW0152665	l-Histidinol phosphate	Organic acid and Its derivatives	0.0011	0.07	down
	MW0104968	Beta-Tyrosine	Benzene and substituted derivatives	0.0102	0.25	down
	MW0108781	N-Formylmethionine	Amino acid derivatives	0.0071	0.53	down
	MW0169518	N-Acetylputrescine	Amines	0.0084	0.37	down
	MW0009415	Phenylacetic acid	Benzene and substituted derivatives	0.0055	0.16	down
Ascorbate and aldarate metabolism	MW0114733	L-Gulose	Sugars	0.0243	1.64	up
	Hmfn000531	Ascorbic acid	CoEnzyme and vitamins	0.0271	5.40	up
a-linolenic Acid, linoleic metabolism	MW0048984	Colnelenic acid	Oxidized lipids	0.0018	16.69	up
	MW0012632	17-Hydroxylinolenic acid	Oxidized lipids	0.0017	0.22	down
	MW0112738	(3Z,6Z)-3,6-Nonadienal	Aldehydes	0.0068	0.54	down
	MW0012346	13(S)-HpOTrE(gamma)	Others	0.1061	0.10	down
	MEDN0784	9-OxoODE	Oxidized lipids	0.0043	0.47	down
	MW0015396	9(S)-HpODE	Oxidized lipids	0.0176	0.73	down
	MEDN1417	(±)13-HpODE	Oxidized lipids	0.0050	0.33	down
Diacylglycerol containing α-linolenic acid	MW0050075	DG(18:1(11Z)/18:2(9Z,12Z)/0:0)	DG	0.0012	3.17	up
	MW0050168	DG(18:2(9Z,12Z)/16:1(9Z)/0:0)	DG	0.0092	1.99	up
Diacylglycerol containing linoleic acid	MW0049810	DG(16:0/18:3(9Z,12Z,15Z)/0:0)	DG	0.0277	1.88	up
	MW0050246	DG(18:3(9Z,12Z,15Z)/18:3(9Z,12Z,15Z)/0:0)	DG	0.0344	1.97	up
	MW0050245	DG(18:3(9Z,12Z,15Z)/18:3(6Z,9Z,12Z)/0:0)	DG	0.0018	4.31	up
	MW0050240	DG(18:3(9Z,12Z,15Z)/16:1(9Z)/0:0)	DG	0.0239	2.58	up
	MW0050726	DG(20:5(5Z,8Z,11Z,14Z,17Z)/18:3(9Z,12Z,15Z)/0:0)	DG	0.0017	2.92	up
	MW0050026	DG(18:0/18:3(9Z,12Z,15Z)/0:0)	DG	0.0030	2.64	up

Fold changes were calculated relative to the S7 diet group (forage mulberry leaf diet).

## Data Availability

The original contributions presented in this study are included in the article.
